# Do You Always Choose What You Like? Subtle Social Cues Increase Preference-Choice Consistency among Japanese But Not among Americans

**DOI:** 10.3389/fpsyg.2017.00169

**Published:** 2017-02-21

**Authors:** Yukiko Uchida, Krishna Savani, Hidefumi Hitokoto, Koichi Kaino

**Affiliations:** ^1^Kokoro Research Center, Kyoto UniversityKyoto, Japan; ^2^Division of Strategy, Management, and Organization, Nanyang Business SchoolSingapore, Singapore; ^3^Koshien UniversityTakarazuka, Japan

**Keywords:** choice-preference consistency, culture, self, social cue, agency

## Abstract

Previous research has suggested that stability of self-concept differs across cultures: in North American cultural contexts, people’s self-concept is stable across social contexts, whereas in Japan, different self-concepts are activated within specific social contexts. We examined the implications of this cultural difference for preference-choice consistency, which is people’s tendency to make choices that are consistent with their preferences. We found that Japanese were less likely than Americans to choose items that they liked the most, showing preference-choice inconsistency. We also investigated the conditions in which Japanese might exhibit greater preference-choice consistency. Consistent with research showing that in Japanese culture, the self is primarily conceptualized and activated by social contexts, we found that subtle social cues (e.g., schematic representations of human faces) increased preference-choice consistency among Japanese, but not among Americans. These findings highlight that choices do not reveal preferences to the same extent in all cultures, and that the extent to which choices reveal preferences depends on the social context.

## Introduction

### Choice and Agency Model across Cultures

People in affluent modern societies make numerous choices in their daily lives, for example, choosing cookies from 85 options in a American supermarket, or choosing from over 100 different types of seafood in a Japanese supermarket. A key question then arises: On what basis do people make their choices? Do they always choose the products what they like the most?

Choice is an agentic behavior that is likely based on people’s internal states, such as their preferences or attitudes. Importantly, past research has shown that making choices based on one’s own preferences is observed more frequently in European-American cultural contexts than in Asian cultural contexts (Iyengar and Lepper, 1999; Savani et al., 2008).

In the current paper, we test cultural differences in preference-choice consistency. We ask whether people in Japan make choices based on their preferences when their sense of agency is activated by subtle social context cues, whereas people in the US choose in accordance with their preferences regardless of the presence or absence of social cues.

### Preference-Choice Consistency across Cultures

Previous research has found that in European Americal cultural contexts, people base their choices on their personal preferences, and are motivated to maintain consistency between their preferences and their choices (Wilken et al., 2011). For example, when asked to choose which of five similar pens to keep, 84% of American participants chose to keep the pen that they had just rated as liking the most (Savani et al., 2008; Study 3). Indeed, in American cultural contexts, good actions “are freely chosen contingent on one’s own preferences, goals, intentions, and motives” (Markus and Kitayama, 2003, p. 7). Individuals in American cultural contexts are highly sensitive to their own internal states, such as their preferences, and make choices as an expression of their self, thus affirming this disjoint model of agency.

Yet, while individuals are defined in terms of core internal attributes in the American cultural contexts (Shweder and Bourne, 1982; Markus and Kitayama, 1991), they are more often defined in terms of social relationships or situations in the Japanese cultural context (Lebra, 1976; Hamaguchi, 1985; Markus and Kitayama, 1991). In Japan, “preferences, goals, and intentions are interpersonally anchored” (Markus and Kitayama, 2003, p. 7). In conjoint models of agency, agentic actions (e.g., choices that are based on personal preferences) are not be salient without a social context. For example, when asked “Who are you?” Japanese respondents were less likely to describe abstract, context-independent attributes, such as personality traits, than Americans. But when asked to answer the same question about a specific social context (e.g., “Who are you at school?”), Japanese were more likely to describe abstract attributes than Americans (Cousins, 1989). Uchida et al. (2009) also suggested that one’s internal states (i.e., emotion) are activated in self-focused situations among Americans with disjoint models of agency, whereas they are activated by social relationship cues (i.e., other persons) among Japanese with conjoint models of agency. Thus, for Japanese people, internal states, including emotions and preferences, are more likely to be activated and expressed when the social context is salient.

### Preference-Choice Consistency across Cultures When the Social Context Is Salient

Importantly, even subtle social cues provide sufficient interpersonal context for the Japanese sense of self to be activated (Kitayama et al., 2004). Thus, we hypothesized that Japanese would exhibit lower baseline preference-choice consistency than Americans, but that under conditions in which the social context is made salient (via subtle social cues), Japanese would exhibit higher preference-choice consistency.

Initial support for the hypothesis that social cues would lead to greater (rather than lower) preference-choice consistency in Japan, comes from previous research manipulating social cues in the standard free choice cognitive dissonance paradigm. In this paradigm, participants are first asked to indicate their preferences for several items. The experimenter then asks them to choose between two closely ranked items, and then reassesses their preferences. The typical finding is that compared to the first round of preference rankings, people increase the ranking of the chosen item and decrease the ranking of the non-chosen item, a phenomenon termed *spreading of alternatives*, which is a way of reducing *cognitive dissonance* (Brehm, 1956).

Although *spreading of alternatives* is a highly reliable finding in the American contexts, multiple studies found that Japanese do not exhibit such choice justification in the standard paradigm (Heine and Lehman, 1997). However, after thinking about the preferences of others, Japanese do spread the alternatives and thus exhibit cognitive dissonance (Kitayama et al., 2004). Similarly, although Japanese do not exhibit cognitive dissonance by spreading the alternatives when making choices for themselves, they show cognitive dissonance when making choices for a friend (Hoshino-Browne et al., 2005).

Furthermore, subtle interpersonal anchors, such as the presence of schematic depictions of human eyes that give a sense that the person is being watched by others (i.e., in a social context) lead Japanese to exhibit cognitive dissonance (Kitayama et al., 2004; Imada and Kitayama, 2010). These findings suggest that making choices for others and making choices in the presence of social cues have similar effects. Indeed, mere exposure to a face cue automatically evokes a social context for those who have a relatively high interdependent self-construal (Qin et al., 2010; Park and Kitayama, 2014).

In the present study, we assessed preference-choice consistency in Japanese and American cultural contexts using a within-person preference-choice paradigm (Savani et al., 2008, Study 3). We employed the same social context cues as used in previous research as they have been shown to automatically evoke a social context (Kitayama et al., 2004; Ishii et al., 2010).

## Materials and Methods

### Participants

We recruited 42 students at Columbia University in the USA (28 women, 14 men; mean age 21.21 years; 15 European American, 4 African American, 2 Latin American, 13 East Asian, 5 South Asian, 2 multiracial, and 1 unreported) and 51 participants at Koshien University and Kwansei Gakuin University in Japan (22 women, 28 men, 1 of unreported gender; mean age 21.12 years). Participants were randomly assigned to either the face (social cue) condition or the no-face (no social cue) control condition. All materials were prepared in English and translated into Japanese for use in Japan. Participants provided written informed consent to participate in this study. Research on human subjects was approved by Columbia University’s Human Subjects protocol IRB-AAAF4543:Y1M00.

### Preference-Rating Phase

In the first round of the study, participants were presented with color images of consumer items, ranging in size from 150 to 240 pixels, at the center of the computer screen one at a time. For each item, they were instructed to “rate the extent to which you like the item” on a scale ranging from 1 (“not at all”) to 4 (“a lot”). Sixty-four different items were presented in random order. The stimuli included eight common everyday items, one from each of eight categories (chairs, colors, cups, tile patterns, plants, shirts, umbrellas, and watches). Within each category, the item images were taken from the same website to create homogenous sets of four items each. There was no manipulation during the preference-rating phase.

### Choice Phase

In the subsequent choice phase, we used the stimuli from the preference-rating round. We divided the eight items from each category into two groups of four items each, yielding a total of 16 choice sets. The order of presentation of the 16 choice sets was randomized for each participant. For each choice set, participants were instructed: “From each group, suppose that you can get one of the four items for yourself. You have to choose one item that you want most for yourself.”

### Social Cue Manipulation

**FIGURE 1:**
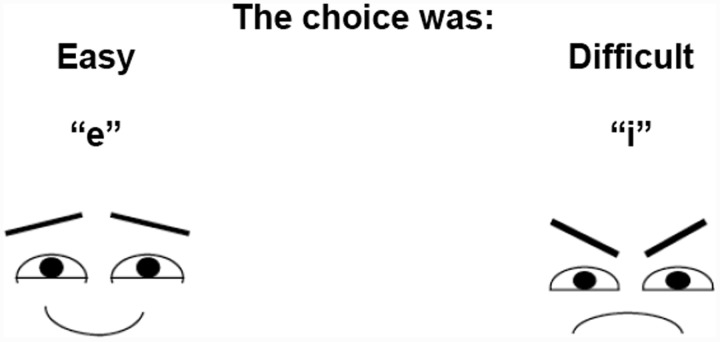
**“Difficulty” screen used in the face condition**.

**FIGURE 2:**
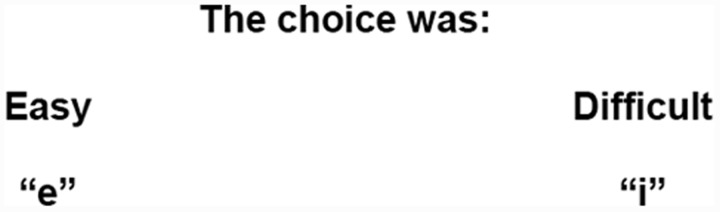
**“Difficulty” screen used in the no-face condition**.

After participants made each choice, they were asked to indicate whether the choice they just made was easy or difficult. In order to manipulate whether social context information was subtly cued or not, participants were randomly assigned to either the face condition or the no-face control condition. In the face condition, participants were shown a schematic smiley face below the “easy” rating and a schematic frowning face below the “difficult” rating (see **Figure 1**; see also Ishii et al., 2010, for a similar manipulation). Thus, participants in the face condition were incidentally exposed to the “eyes of the other” after every trial of the choice round. In order to present face cues immediately prior to the first choice trial, we also presented these schematic faces on the instruction screen used to explain to participants that they would be asked to judge the difficulty of their choice after each trial. In the no-face control condition, participants were not shown any schematic images with the difficulty questions (see **Figure 2**).

## Results

We computed the consistency between participants’ preference ratings and their subsequent choices, following the procedure used by Savani et al. (2008; Study 3). To control for heterogeneity in mean preference ratings across choice sets, we converted participants’ preference ratings of the four items within each choice set into ranks, with ranks averaged across trials. For ease of interpretation, we reversed the rank of the chosen item, such that “4” indicated that the most preferred item was chosen and “1” indicated that the least preferred item was chosen. For example, if participants rated the four items in a choice set 4, 3, 2, and 1, then the corresponding ranks would be 4, 3, 2, and 1. If they rated the four items 4, 4, 4, and 3, then the corresponding ranks would be 3, 3, 3, and 1. If they rated the four items, 2, 2, 2, and 1, then the corresponding ranks would still be 3, 3, 3, and 1. This ranking procedure removes between-participant and between-trial differences in mean ratings of the items.

**FIGURE 3:**
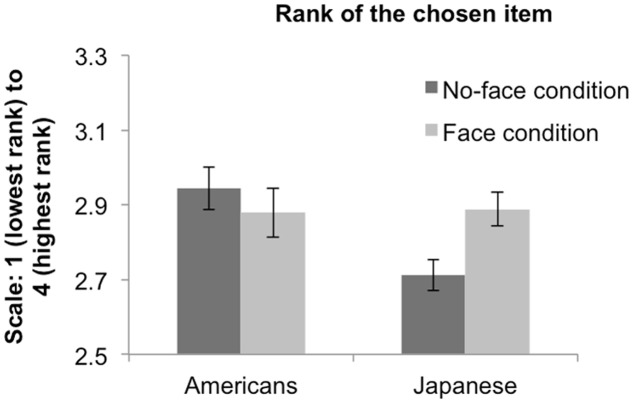
**Mean rank of the chosen item by culture and condition (adjusted marginal means)**. Higher numbers indicate greater preference-choice consistency. Error bars indicate the standard errors of the means.

For each trial in the choice phase, we calculated the rank of the chosen item. A higher rank for the chosen item indicates greater preference-choice consistency. We averaged participants’ rankings of the chosen items across the 16 trials to yield a composite measure of preference-choice consistency, and submitted this measure to a 2 (Culture) × 2 (Condition) ANOVA. We found a main effect of culture, *F*(1,89) = 4.56, *p* = 0.035, indicating that overall, Americans showed a higher preference-choice consistency than Japanese, *M*_Americans_ = 2.91, *SE* = 0.043, *M*_Japanese_ = 2.80, *SE* = 0.033. The main effect of condition was not significant, *F*(1,89) = 1.17, *p* = 0.280, but there was a significant Culture × Condition interaction, *F*(1,89) = 5.38, *p* = 0.023. Follow-up contrasts indicated that there was no significant effect of condition for Americans, *F*(1,89) = 0.70, *p* = 0.410, *M*_Face_ = 2.88, *SE* = 0.066, *M*_No-face_ = 2.94, *SE* = 0.057, but Japanese participants had significantly higher preference-choice consistency in the face condition than in the no-face condition, *F*(1,89) = 6.41, *p* = 0.01 *M*_Face_ = 2.89, *SE* = 0.045, *M*_No-face_ = 2.71, *SE* = 0.042. Further, whereas Japanese participants showed lower preference-choice consistency than American participants in the no-face condition, *F*(1,89) = 9.76, *p* = 0.002, this difference was not significant in the face condition, *F*(1,89) = 0.02, *p* = 0.900 (see **Figure 3**).

We next tested whether the difficulty of choices varied across culture and condition. We submitted the proportion of trials that participants indicated as being “difficult” to a 2 (Culture) × 2 (Condition) ANOVA. There was a significant main effect of culture, *F*(1,89) = 14.85, *p* < 0.001, *M*_Americans_ = 0.37, *SE* = 0.023, *M*_Japanese_ = 0.49, *SE* = 0.022. This result is consistent with the notion that contemporary American society has many more opportunities to perceive and experience actions as choices (Snibbe and Markus, 2005; Savani et al., 2010) compared to Japanese society. However, neither the main effect of condition nor the Culture × Condition interaction were significant, *p*’s > 0.330. The absence of any interaction effect indicates that the social cue did not influence the difficulty of the choice itself. Thus, subjective perceptions of choice easiness was independent from preference-choice consistency

To test the robustness of the findings, we conducted another ANOVA with preference-choice consistency as the dependent measure, culture and condition as predictors, and participants’ gender, age, and proportion of difficult trials as covariates. The key Culture × Condition interaction remained significant, *F*(1,85) = 6.26, *p* = 0.010, indicating that the observed interaction effect was robust even after gender, age, and difficulty of the choice trials were controlled for.

## Supplemental Study

Although the data supported our theoretical prediction that social cues can activate internal states and increase preference-choice consistency among Japanese participants, there is an alternative explanation: instead of activation of Japanese agentic state of mind, the social cues might have Japanese to focus on social norms and others’ expectations (Savani et al., 2015). We tested these two explanations in a supplemental survey study.

### Method

437 Japanese participants recruited through the task recruitment website Lancers and 425 Americans recruited through Amazon Mechanical Turk completed an online survey. We analyzed 376 Japanese (mean age = 38.20; 55% female) 372 Americans (mean age = 34.88; 53% female; 78.0% European Americans, 6.4% African Americans, 3.8% Latino Americans, 3.6% Native Americans, 5.1% Asian Americans, 3.6% other ethnicity). Selection criteria required that participants correctly answer at least 50% of eight attention check questions, be either US-born US citizens (in the US sample) or JP-born Japanese citizens (in the Japanese sample) and be aged 20–75 to be included for analysis.

We presented participants with five items on seven-point Likert scale (1 = strongly disagree, 7 = strongly agree) measuring their “preference-choice consistency orientation” (PCC, e.g., “When making choices, I believe it is important to express my preferences/attitudes”) and five measuring their “social concerns about preference-choice consistency” (SCC, e.g., “I will lose my face if I do not act consistent with my opinions in front of others”). Internal reliability of the PCC scale was α = 0.65 (JP) and α = 0.73 (US), and that of the SCC scale was α = 0.72 (JP) and α = 0.77 (US). The correlation between PCC and SCC scales was *r* = 0.36 (*p* < 0.001) in Japan and *r* = 0.42 (*p* < 0.001) in the US.

### Results and Discussion

We conducted a 2 (JP and US) *×* 2 (PCC and SCC) repeated measures ANOVA and found a significant main effect of culture [*F*(1,751) = 155.80, *p* < 0.0001], showing that Americans showed relatively higher PCC (*M* = 4.86; *SE* = 0.047) and SCC (*M* = 4.95, *SE* = 0.050) than Japanese (PCC: *M* = 4.49, *SE* = 0.047; SCC: *M* = 3.89, *SE* = 0.050). We also found a significant interaction [*F*(1,751) = 83.93, *p* < 0.0001], suggesting that differences across cultures on PCC was smaller than differences across cultures on SCC. That is, SCC was lower than PCC in Japan, but SCC was higher than PCC in the US. We interpret these findings to suggest that Japanese are not concerned if their choices are inconsistent with their preferences in front of others. They also suggest that Study 1’s findings with Japanese participants is unlikely to be due to social concerns and is instead more likely due to the activation of a sense of self that values preference-choice consistency.

## General Discussion

The present study found that people in Japanese cultural contexts were less likely to make choices according to their preferences than people in American cultural contexts, and that exposure to subtle social cues during the choice phase increased Japanese participants’ preference-choice consistency, but not that of American participants. This indicates that in cultural contexts in which the self is assumed to exist primarily in an interpersonal context (not autonomously by itself), social cues can also direct attention toward the self and its internal states, thus leading people to base their choices on their preferences. The present findings highlight the importance of the presumed other for even seemingly trivial and private acts, such as choosing which cup, umbrella, or shirt one would like to have, consumer choices that are very common both in the US and in Japan.

Unlike previous research findings that Americans are less likely to justify their choices after exposure to social cues (Kitayama et al., 2004), in this study Americans’ preference-choice consistency was unaffected by exposure to social cues. This might be due to a ceiling effect, arising from Americans predominantly basing their choices on their preferences (Savani et al., 2008). Future research can further examine the differential effects of exposure to social cues on post-choice justification and preference-choice consistency.

The present finding that subtle social cues increase Japanese people’s preference-choice consistency contrasts with recent research showing that subtle social cues decrease preference-choice consistency for Indians (Savani et al., 2015). In India, preferences appear to be salient under normal conditions and are deactivated in the presence of social cues, whereas in Japan, preferences appear to be particularly obscure under normal conditions, but are activated in the presence of social cues. Thus, the relationship between the contingency of the self on the social context varies significantly across Japan and India.

Although Indian and Japanese cultures are both considered interdependent cultures, one might expect important cultural differences to exist when it comes to agency and social relationships. Based on Hofstede’s analysis (Hofstede, 2001), Indian cultural contexts are higher on power distance compared to Japanese cultural contexts. In India, choices are perhaps more likely to express “social conformity,” and thus need to be consistent with the expectation of respected others, such as one’s parents (Savani et al., 2012). In addition, research suggests that Indians’ personal preferences are usually salient, even in non-social contexts (e.g., Sen, 2005). When exposed to social cues, however, their preferences shift from the self to others given the importance of collectivism and social conformity. Thus, in the Indian cultural context, social relationship cues might be a distractor from self-expressive emotional states (such as focusing on choice). Instead, personal preferences might be suppressed and social adjustment toward others might be salient. As a result, Indians’ preference-choice consistency might be reduced, particularly if Indians are motivated to connect with others rather than to distinguish themselves from others (Brewer, 1991; Kwan et al., 2015).

Another possibility is that the discrepancy between personal preferences and social norms might differ across Indian and Japanese contexts. Given that social norms are stronger in India than in Japan (Gelfand et al., 2011), people’s personal preferences might be more likely to deviate from social norms in India. Further, if social cues activate normative concerns in India (Savani et al., 2012), people’s personal preferences might further diverge from perceived norms. This might explain why Indians’ behaviors (such as choices) in social contexts are more consistent with the expectation of others and further from their personal preferences. In Japan, if people’s personal preferences are not as distant from perceived norms as in India, even if social cues activate normative concerns, these might not push people away from their preferences. This might explain why Japanese people’s choices in social contexts are more consistent with their personal preference. Future research can directly test these ideas by assessing the ease with which Indians and Japanese describe their preferences, and by measuring the distance between personal preferences and perceived norms, in both social and non-social contexts.

In addition to contributing to social psychological theories on choice, our findings have implications for cross-cultural consumer behavior. Most advertising and marketing campaigns attempt to change individuals’ preferences for different consumer objects. But if people are not very likely to choose according to their own personal preferences in the absence of social cues, these strategies might have limited success. Our findings suggest that marketers may benefit from creating conditions that cause consumers to make choices in a social context.

Our findings provide a more nuanced view of the literature on cultural differences in self-consistency (Kanagawa et al., 2000; Suh, 2002). They suggest, perhaps ironically, that Japanese might exhibit greater cross-situational self-consistency if their self-evaluations are made *in a social context* (not just made *about a social context*). Although Japanese are more likely to describe themselves differently across different social situations, when their immediate self is interpersonally anchored by even minimal social cues, their self-view might become more consistent across different social situations.

One limitation of this paper is that we could not elucidate the mechanism underlying the effect of social cues on preference-choice consistency. We assume that increased attention to incongruities between different aspects of the self is responsible for the effect, but the process is likely implicit and unconscious. One might argue that the schematic eyes in our study increased preference-choice consistency among Japanese by reminding participants to attend closely to the task rather than choosing randomly. Yet, past research on cognitive dissonance suggests that this is an unlikely possibility. In the presence of the schematic eyes, Japanese exhibited a greater divergence between their current and their previously stated preferences (Kitayama et al., 2004), whereas more attentive preference ratings should lead to a smaller discrepancy between previously stated and current preferences. However, future research can clarify the process by identifying how heightened attention to self-incongruities acts as a mediator.

Whereas economists and researchers on decision making typically assume that choices reveal either pre-existing preferences (von Neumann and Morgenstern, 1944) or constructed preferences (Bettman et al., 1998), our findings show that the extent to which choices reveal preferences varies across cultures and contexts, and that preferences, whether pre-existing or constructed, influence choices in ways that are culturally contingent. The current study suggests that choice is not only a personal or individualized behavior, but is also determined by social relationships, especially in the Japanese social context.

## Author Contributions

YU and KS contributed equally to all the work (design, data collection, analysis, writing). HH and KK also contributed equally to the research design and data collection work.

## Conflict of Interest Statement

The authors declare that the research was conducted in the absence of any commercial or financial relationships that could be construed as a potential conflict of interest. The reviewer NS and handling Editor declared their shared affiliation, and the handling Editor states that the process nevertheless met the standards of a fair and objective review.
